# WRANet: wavelet integrated residual attention U-Net network for medical image segmentation

**DOI:** 10.1007/s40747-023-01119-y

**Published:** 2023-06-08

**Authors:** Yawu Zhao, Shudong Wang, Yulin Zhang, Sibo Qiao, Mufei Zhang

**Affiliations:** 1 School of Computer Science and Technology, China University of Petroleum, Qingdao, Shandong China; 2 College of Mathematics and System Science, Shandong University of Science and Technology, Qingdao, Shandong China; 3 Inspur Cloud Information Technology Co, Inspur, Jinan, Shandong China

**Keywords:** Medical image segmentation, Discrete wavelet transform, Downsampling, Residual attention module, Aneurysm image

## Abstract

Medical image segmentation is crucial for the diagnosis and analysis of disease. Deep convolutional neural network methods have achieved great success in medical image segmentation. However, they are highly susceptible to noise interference during the propagation of the network, where weak noise can dramatically alter the network output. As the network deepens, it can face problems such as gradient explosion and vanishing. To improve the robustness and segmentation performance of the network, we propose a wavelet residual attention network (WRANet) for medical image segmentation. We replace the standard downsampling modules (e.g., maximum pooling and average pooling) in CNNs with discrete wavelet transform, decompose the features into low- and high-frequency components, and remove the high-frequency components to eliminate noise. At the same time, the problem of feature loss can be effectively addressed by introducing an attention mechanism. The combined experimental results show that our method can effectively perform aneurysm segmentation, achieving a Dice score of 78.99%, an IoU score of 68.96%, a precision of 85.21%, and a sensitivity score of 80.98%. In polyp segmentation, a Dice score of 88.89%, an IoU score of 81.74%, a precision rate of 91.32%, and a sensitivity score of 91.07% were achieved. Furthermore, our comparison with state-of-the-art techniques demonstrates the competitiveness of the WRANet network.

## Introduction

Intracranial aneurysm (IAS) is a common disease with a high mortality rate, and timely and accurate identification and treatment are essential for patients [1]. In medical imaging, computed tomography and magnetic resonance angiography are convenient, effective, and reliable methods for detecting IAS. However, professionally trained physicians are required to analyze and interpret them. Undoubtedly, this will be very time consuming and increase the burden on doctors [2, 3]. Therefore, a robust and reliable artificial intelligence algorithm model is urgently needed to improve this problem.

Medical image segmentation algorithms have been a hot problem for research, which is a practical guide to facilitate pathological assessment and subsequent disease diagnosis and treatment and a very challenging task [4]. Traditional medical image segmentation methods usually use features such as grayscale values, shapes, and textures to segment images; however, the best segmentation effect cannot be achieved by relying on pixels and contours alone. In recent years, with the rapid rise of artificial intelligence technology, the emergence of deep learning techniques has widely advanced medical image segmentation, such as brain tumor segmentation [5, 6], skin damage segmentation [7], aneurysm segmentation [8, 9], and ventricular segmentation [10]. Compared with traditional methods, it can capture meaningful contextual information in images, which leads to more accurate diagnosis and segmentation, laying the foundation for further development of medicine [11].

In the field of computer vision, some advanced network structures have been proposed, such as FCN [12], SegNet [13], Deeplabv3+ [14], GoogleNet [15], Residual network [16], and DenseNet [17]. Most of these network structures are improved on the CNN structure [18] and have achieved excellent performance. Compared with CNN methods, FCN methods can classify images at the pixel level while preserving the spatial information of the original image and improving computational efficiency. SegNet consists of an encoder, decoder, and pixel-level classification layers that can independently compute the class probabilities of pixels. Deeplabv3+ improves Xception [19] and depth-separated convolution to improve the semantic performance of the segmentation task. GoogleNet uses the Inception module to break the limits of network depth and width to achieve deeper feature extraction and multi-scale feature processing. Skip connections are used for the Residual block in the Residual network to alleviate the gradient vanishing and network degradation associated with increasing depth in deep neural networks. DenseNet establishes dense connections at shallow and deep layers to improve model performance by enabling feature reuse through feature channel connections.

However, when dealing with medical image segmentation tasks, the U-Net model reduces errors in feature extraction by connecting the encoder and decoder through the skip layer and has achieved great success in medical image segmentation [20]. Inspired by the Residual network, He et al. [21] proposed a Residual learning framework integrated into the U-Net model to extract the features of the deeper network. Zhang et al. [22] proposed a residual context network (ConResNet) to improve the accuracy of pancreatic and brain tumor segmentation. In addition, the proposed attention module was initially used for image classification [23] and recently also used for medical image segmentation [24, 25]. Gu et al. [7] proposed a scale attention module to obtain multiple-scale feature maps. Yu et al. [26] constructed a six-layer residual neural network to fully extract the features of mechanical vibration signals and visualize them using gradient and feature vector-based class activation maps. Cao et al. [27] proposed a transformer-based U-shaped encoder–decoder structure called Swin-Unet, which fuses the extracted contextual features with the multi-scale features of the encoder through a jump connection to compensate for the spatial loss caused by downsampling. Xu et al. [28] proposed a novel adversarial discriminative network (segAN) with a multi-scale L1 loss function that forces the critic and segmenter to learn to capture both global and local features of long- and short-range spatial relationships between pixels simultaneously, outperforming state-of-the-art methods in terms of Dice scores and accuracy.

More importantly, recent studies show that common image noise will affect the networks judgment of final results to a certain extent, proving the networks weak robustness [29, 30]. Robustness is closely related to standard downsampling methods such as max pooling and average pooling. The traditional downsampling technique cannot achieve the effect of denoising and cannot improve the robustness of the model. In signal processing, wavelet analysis is joint image compression and denoising technology, which can separate the low-frequency and high-frequency data information (some image noise). It has been widely applied in the fields of image processing [31, 32] and signal processing [33, 34].

Inspired by the above work, we proposed a residual attention network model (WRANet) based on wavelet integration to suppress noise propagation, gradient vanishes, and network feature reduction. During the downsampling period, low-frequency information propagates through the network to obtain higher-level features, while high-frequency information is discarded as noise. We used different discrete wavelets to remove image noise and evaluate the Dice coefficient(Dice), intersection over union(IoU), precision (PR), and sensitivity (SE) of the WRANet network on four data sets.

Our main contributions are summarized as follows:

1. In the encoder, we proposed a new downsampling module with different wavelet functions instead of pooling layers for downsampling to extract multi-scale tumor features and remove noise efficiently.
2. A jump connection layer is used between the encoder and decoder, and a residual attention model is incorporated to mitigate the performance degradation caused by gradient disappearance and feature information loss.
3. In this paper, we tested on the aneurysm and polyp datasets and demonstrated through experimental results that WRANet achieves better segmentation performance and is a very effective strategy.

## Related work

### Wavelets application

Wavelet analysis is widely used in signal analysis, image processing, medical imaging and diagnosis, seismic exploration data processing, etc. In image processing, discrete wavelet transform (DWT) is usually used to decompose 2D images [35]. DWT decomposes the image into LL, LH, HL, and HH subbands through high-low pass filters. Among them, LL represents the low-frequency coefficient, representing the primary structural information of the picture. At the same time, LH, HL, and HH are the high-frequency coefficients, describing the details of horizontal, vertical, and diagonal coefficients, respectively.

In recent work, wavelets have also been integrated into deep learning models for image reconstruction [36], downsampling operation [37], and noise suppression [38]. For example, in [39], the author proposed a new wavelet hybrid network (WH-NET) for single image defogging and DWT as a feature extraction layer to achieve a multilevel representation of fuzzy images. Liu et al. [40] proposed a novel multilevel wavelet convolutional neural network model (MWCNN), which introduced wavelet transform to reduce the size of feature images and reconstructed high-resolution feature images using inverse wavelet transform. Verma et al. [41] proposed a wavelet-based convolutional neural network architecture to detect SARS-NCOV, using mother wavelet functions from different families to perform discrete wavelet transform (DWT) and two-stage DWT decomposition to suppress the noise in chest X-ray images. Kang et al. [42] proposed a residual wavelet network, which synergistically combined the expression ability of deep learning with the denoising performance of the wavelet framework. Ma et al. [43] used a trained iWave++ wavelet transform as a new end-to-end method for optimizing images with lossy iWave++ to achieve state-of-the-art compression efficiency compared to deep network-based methods. Huang et al. [44] proposed a wavelet-inspired reversible network (WINNet) by combining wavelet and neural network-based methods to construct a denoising of the sparse coding process, thus recovering the noisy image to a clean one. In our work, we use different wavelet functions to replace the max-pooling layer for downsampling operation, aiming to eliminate the noise caused by the upper-layer network and extract tumor multi-scale image features to improve the interpretability of the network.The experimental results confirmed that it improved the performance and retained the details and texture features of the original image.

### Attention mechanism

The attention mechanism is widely used in deep learning models. It originates from human research on vision and focuses on multiple details by generating context vectors. It has been applied to different scenes, such as text translation [45], image description [46], and speech recognition [47], and achieved great success.

Due to the excellent performance of the attention mechanism, it is gradually applied to medical segmentation tasks. Hu et al. [48] proposed a dense convolutional network with a mixed attention mechanism to calibrate feature maps from the upper layer using channel and spatial attention. Wang et al. [49] proposed a mixed dilated attentional convolution (HDAC) framework for liver tumor segmentation to fuse information from receptive fields of different sizes. Poudel et al. [50] used a compound-scaled EfficientNet to capture multi-scale global features to resolve limited long-range feature dependencies while exploiting an attention mechanism to suppress noisy and useless features. Zhuang et al. [51] designed a multi-mode cross-latitude attention (MCDA) module to automatically capture valid information from all dimensions of the multi-mode image, achieving excellent segmentation performance in the CEREBRO spinal fluid region. More importantly, we proposed a residual attention network model (RAM), which is used to establish skip connections and improve the context information fusion between encoder and decoder to effectively utilize the context information and the characteristics of the region of concern.

## Our method

### Overview

**Fig. 1:**
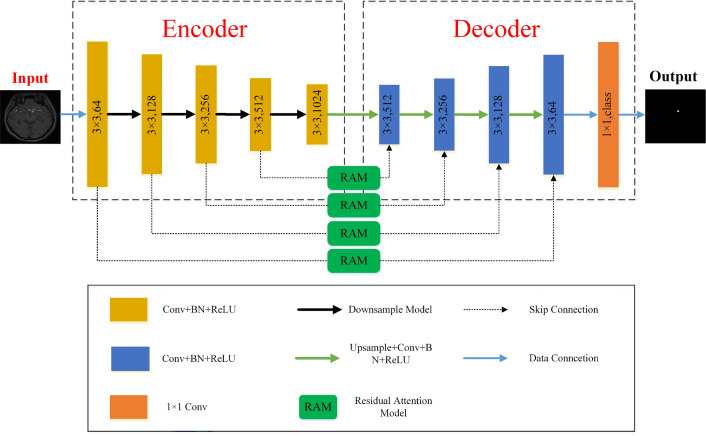
Our proposed wavelet residual attention network (WRANet). $1 \times 1$ and $3\times 3$ represent the size of the convolution kernel, while 64, 128, 256, 512, and 1024 represent the number of output channels. We use 4 downsampling modules, 4 upsampling modules, and 4 residual attention modules

The WRANet model mainly comprises an encoder, decoder, and RAM module. We proposed a new downsampling strategy to mine more compelling features in the image, which used a two-dimensional discrete wavelet transform (DWT) instead of the maximum pooling layer to extract features and remove noise. The decoder integrates the image information extracted by the encoder in the decoder. The bilinear interpolation method is used to restore the image information to reduce the loss of image features. We proposed a residual attention module (RAM) to use better image features, which can automatically focus on areas with significant features while ignoring irrelevant sites during training. Meanwhile, the residual structure can alleviate the problems of feature loss, gradient explosion, and network degradation. Figure 1 shows our proposed WRANet architecture.

In this work, we use bold letters and letters to denote matrices and scalars, e.g., input image ${\textbf {X}}$, residual attention module output ${\varvec{{x}}^o}$, and wavelet transform functions $\psi \left( x \right)$, and $\mathop \varphi \limits ^ \sim \left( x \right)$, etc.

**Fig. 2:**
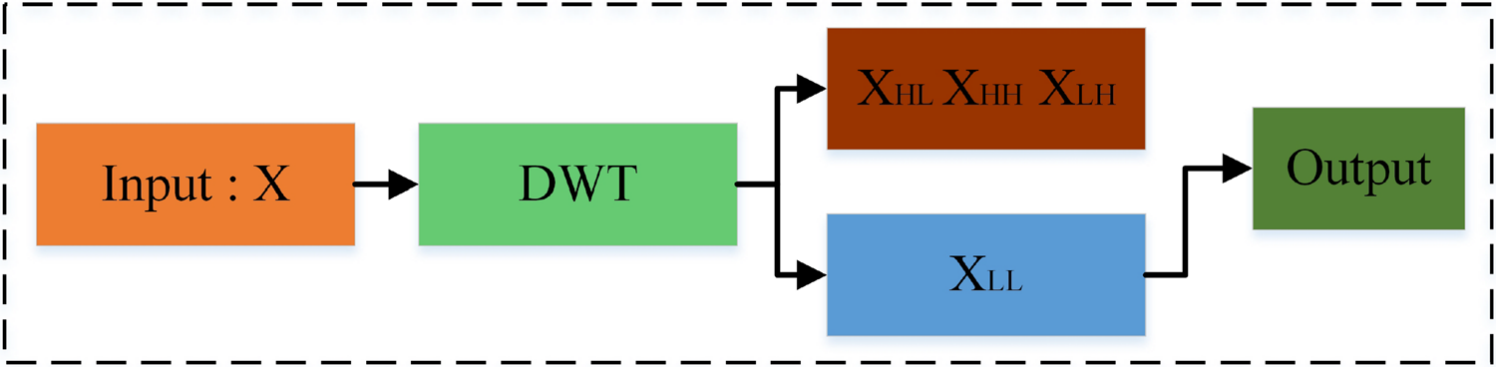
Details of downsampling module (DWT represents discrete wavelet transform, wavelet subsampling in the WRANet network filters out noise and facilitates information propagation)

### Downsampling module

Figure 2 shows the details of the downsampling module. We designed a discrete wavelet transform layer for feature sampling and applied it to improve the performance of the deep neural network for aneurysm image segmentation. Max pooling is a standard downsampling method in a deep neural network, but it is easy to destroy the structure information of the feature graph. Therefore, we integrate a 2D discrete wavelet transform into the network to replace the max-pooling layer, which can extract features more effectively and remove unnecessary noise information. The subsampled module we have rewritten can be adapted to various orthogonal and biorthogonal wavelets, such as Haar, Daubechies, Symlets, biorthogonal, and reverse biorthogonal. We first introduce the basic theory of 2D discrete wavelet transform.

2D discrete wavelet transform is closely related to scale function $\varphi \left( x \right)$, and wavelet function $\psi \left( x \right)$, which form the stable basis of signal space *R*. In discrete wavelet transform, scale and wavelet function correspond to low-pass filter $l = {\left\{ {{l_i}} \right\} _{i \in z}}$, and high-pass filter $h = {\left\{ {{h_i}} \right\} _{i \in z}}$, and low-pass and high-pass filter decompose data to obtain low-frequency and high-frequency information. Haar, Daubechies, and Symlets are orthogonal wavelets. The corresponding $l = {\left\{ {{l_i}} \right\} _{i \in z}}$ value varies with the corresponding wavelet scale *S*. We choose the size of the scale *S* is $1 \le S \le 7$, when $S=1$ is Haar wavelet. Its filter length is 2*S*, while the high-pass filter can be defined as:

1 $$\begin{aligned} {h_i} = {\left( { - 1} \right) ^i}{l_{2n + 1 - i}}, \end{aligned}$$

where, $n\in \left\{ 0,1,2,3... \right\}$, *i* denotes the size of the filter, *z* denotes a positive integer.

Biorthogonal and reverse biorthogonal wavelets are biorthogonal. If the two dual wavelet functions $\psi \left( x \right)$, and $\mathop \psi \limits ^ \sim \left( x \right)$ satisfy:

2 $$\begin{aligned} {\psi _{m,n}}\left( x \right) ,\mathop {{\psi _{j,k}}}\limits ^ \sim \left( x \right) = \delta \left( {m - n} \right) \delta \left( {n - k} \right) , \end{aligned}$$

Then $\psi \left( x \right)$, and $\mathop \psi \limits ^ \sim \left( x \right)$ are biorthogonal, and the corresponding scale functions and must also satisfy:

3 $$\begin{aligned} {\varphi _{j,m}}\left( x \right) ,\mathop {{\varphi _{j,n}}} \limits ^ \sim \left( x \right) = \delta \left( {m - n} \right) , \end{aligned}$$

**Fig. 3:**
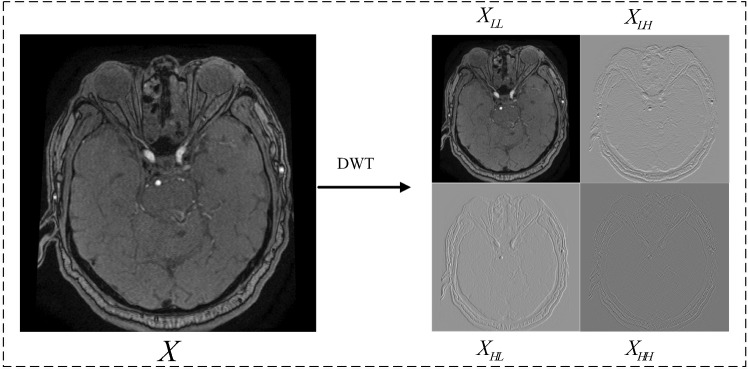
2D discrete wavelet transform decomposes aneurysm image

where *j* represents the scale of wavelet. Then $\psi \left( x \right)$ and $\mathop \varphi \limits ^ \sim \left( x \right)$ are a pair of orthogonal wavelet bases, and there is orthogonality between them. After constructing the biorthogonal wavelet, the original two basis functions are changed into four. Accordingly, *l*, $\mathop l\limits ^ \sim$, *h*, and $\mathop h\limits ^ \sim$ together constitute orthogonal filter banks, and filter banks can be used for image reconstruction. Similarly, we choose the size of scale *S* as $1 \le S \le 4$, and different scales correspond to different values of $l = {\left\{ {{l_i}} \right\} _{i \in z}}$ of the low-pass filter. A high-pass filter can be defined as:

4 $$\begin{aligned}{} & {} {h_i} = {\left( { - 1} \right) ^i}{\mathop {l_{2n+1-i}}\limits ^ \sim }, \end{aligned}$$

5 $$\begin{aligned}{} & {} {\mathop {h_{i}}\limits ^ \sim } = {\left( { - 1} \right) ^i}{l_{2n + 1 - i}}, \end{aligned}$$

2D-DWT of the image can be described as follows: first, 1D-DWT is performed on each row of the image to obtain the low-frequency component $\mathrm L$ and high-frequency component $\mathrm H$ of the original image in the horizontal direction; second, 1D-DWT is performed on each column of the obtained data to obtain the low-frequency component $\mathrm LL$ of the original image in the horizontal and vertical directions. Low frequency in the horizontal direction and high frequency in the vertical direction $\mathrm LH$, high frequency in the horizontal direction and vertical direction $\mathrm HL$, and low frequency in the horizontal and vertical direction $\mathrm HH$. Given 2D image **X**, its 2D-DWT can be defined as:

6 $$\begin{aligned} \left\{ \begin{array}{l} {{\textbf {X}}_{\textrm{LL}}} = l{\textbf {X}}{l^T},\\ {{\textbf {X}}_{\textrm{LH}}} = l{\textbf {X}}{h^T},\\ {{\textbf {X}}_{\textrm{HL}}} = h{\textbf {X}}{l^T},\\ {{\textbf {X}}_{\textrm{HH}}} = h{\textbf {X}}{h^T}, \end{array} \right. \end{aligned}$$

After the input image **X** executes the lower sampling block, its output consists of four parts. Where ${{\textbf {X}}_{\textrm{LL}}}$ is the low-frequency component, representing the primary characteristic information of the image; ${{\textbf {X}}_{\textrm{HL}}}$, ${{\textbf {X}}_{\textrm{HH}}}$, and ${{\textbf {X}}_{\textrm{LH}}}$ are the three high-frequency components, representing the vertical, diagonal, and horizontal detail components of the input data **X**, respectively, which reflect the noise of the image.

In training the network, we discard the high-frequency information of the image and only keep the low-frequency information for transmission in the network. Taking the Haar wavelet as an example, Fig. 3 shows the decomposition process of the 2D discrete wavelet for aneurysm images.

### Residual attention module

**Fig. 4:**
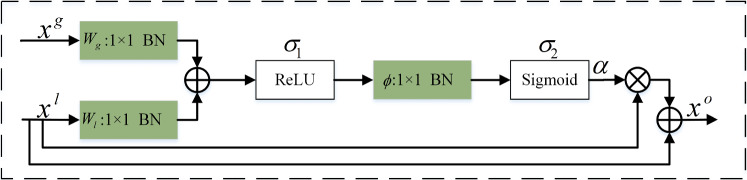
Residual attention module

In our network, the design of the residual attention module is inspired by AG [24], which used an attention gate to recalibrate the feature graph. In addition, the addition of residual structure also avoids feature loss, as shown in Fig. 4.

**Fig. 5:**
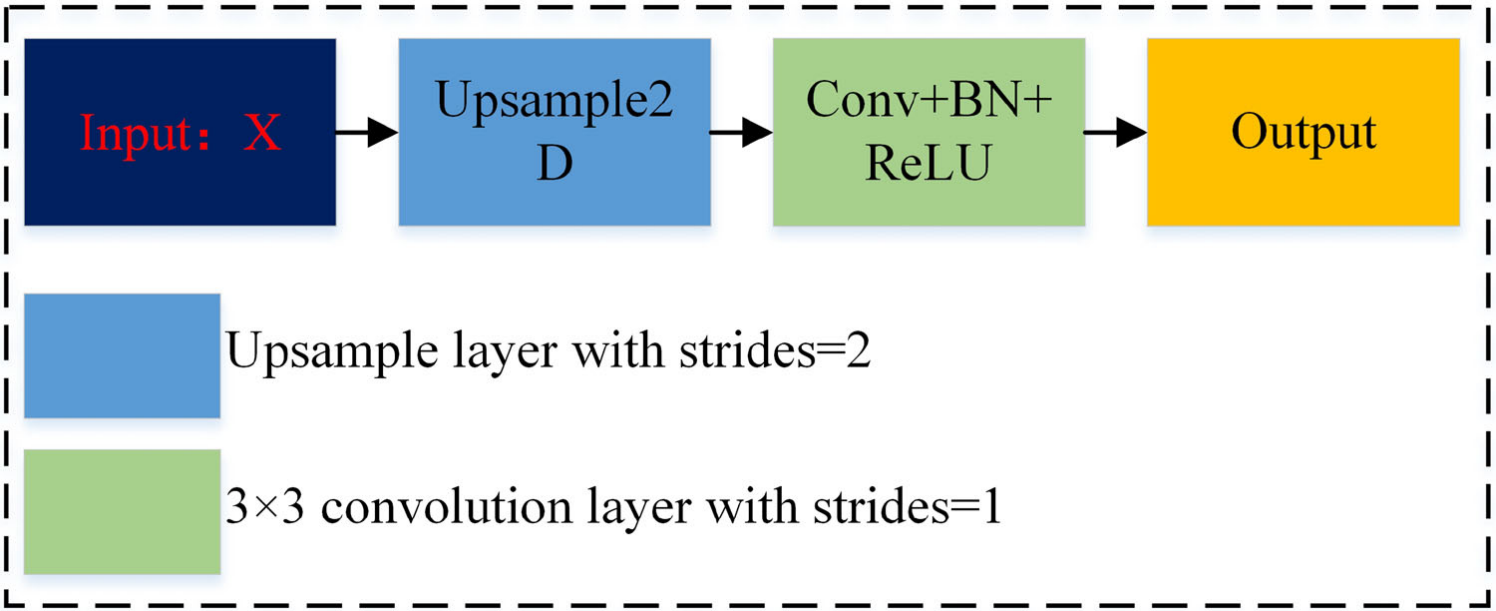
Upsampling model structure

Let ${\varvec{x}^g}$ represent the advanced features from the decoder, the size of the input feature graph is $C \times H \times W$, ${\varvec{x}^l}$ is from the low-level features of the encoder, and the size of the input feature graph is $C \times H \times W$, where *C* represents the feature channel, *H* and *W* represent the height and width of the image, respectively. First, we used $1 \times 1$ convolution to reduce the dimension of the input feature graph of the two parts so that the number of channels is the same. Then after the batch normalization layer (to ensure relatively stable data distribution and accelerate network convergence), the results of the two parts are added together, and the ReLU activation function is performed (to increase the nonlinear relationship between each layer). One-dimensional convolution is used to reduce the number of feature channels to 1. After the Sigmoid function, the pixel-level attention coefficient $\alpha$ is obtained and multiplied by ${\varvec{x}^l}$ to obtain the feature map after calibration. The attention coefficient is as follows:

7 $$\begin{aligned} \alpha = {\sigma _2}\left( {{\varvec{\phi } ^T}\left( {{\sigma _1}\left( {{\varvec{x}^g}\varvec{W}_g^T + {\varvec{x}^l}\varvec{W}_l^T} \right) } \right) } \right) , \end{aligned}$$

For a given matrix $\varvec{\phi }$, *T* denotes its transpose. ${\sigma _1}$ represents ReLU function, and ${\sigma _2}$ represents Sigmoid function so that the value of $\alpha$ falls in (0,1). $\alpha$ assigned different weights to each pixel feature to ensure the interaction between all feature image pixels. Therefore, the recalibrated feature graph $\mathop {\varvec{x}}\limits ^ \wedge$ is expressed as:

8 $$\begin{aligned} \mathop {\varvec{x}}\limits ^ \wedge = \alpha \cdot {{\varvec{x}}^l}, \end{aligned}$$

where the number of output channels of $\mathop {\varvec{x}}\limits ^ \wedge$ is *C*, and its value is 64, 128, 256, and 512, respectively, at different convolution layers of the encoder. In addition, the residual connection is used to improve information transmission during network training, and the output is as follows:

9 $$\begin{aligned} {{\varvec{x}}^o} = \mathop {\varvec{x}}\limits ^ \wedge + {{\varvec{x}}^l}, \end{aligned}$$

### Upsampling model

Figure 5 shows the details of the upsampling module. In this work, the bilinear interpolation method is used to enlarge the feature graph, and $3\times 3$ convolution with a step size of 1 and ReLU function can increase the nonlinear expression ability of the network. Therefore, the primary purpose of the upsampling module proposed is to avoid feature loss and improve the network’s performance.

In the decoding process, after each layer of convolution, the number of channels in the feature graph will be reduced by half, and after the upsampling module, the size of the feature graph will be twice that of the previous layer. Finally, the network output will be changed to the original input size. We let **X** be the input of the module so that the output ${\varvec{x}_{h + 1}}$ can be defined as:

10 $$\begin{aligned} {\varvec{x}_{h + 1}} = \textrm{BR}\left( {\varvec{C}_{3 \times 3}^1\left( {{\varvec{U}^2}\left( {{\varvec{x}_h}} \right) } \right) } \right) , \end{aligned}$$

where $\varvec{C}$ represents the $3 \times 3$ convolution layer with a step size of 1, ${\varvec{U}^2}$ represents the upsampling layer with a step size of 2, and $\mathrm BR$ represents the BN layer and the ReLU activation layer.

### Hybrid loss function

In the medical image segmentation task, the loss function comprises classification and segmentation loss [52]. In this paper, to improve the segmentation performance of medical images, the cross-entropy loss function and Dice loss function are combined as the loss function of this paper. Cross-entropy loss describes the distance between the predicted value and ground truth, while Dice loss measures the degree of consistency between the predicted value and ground truth. Cross-entropy loss ${L_{\textrm{BCE}}}$ and Dice loss function ${L_{\textrm{Dice}}}$ are shown below:

11 $$\begin{aligned}{} & {} {L_{\textrm{BCE}}}\mathrm{{ = }} - \frac{1}{n}\sum \limits _{i = 1}^n {\left[ {{y_i} \cdot \log \left( {{p_i}} \right) + \left( {1 - {y_i}} \right) \log \left( {1 - {p_i}} \right) } \right] } ,\nonumber \\ \end{aligned}$$

12 $$\begin{aligned}{} & {} {L_{\textrm{Dice}}}\mathrm{{ = 1}} - \frac{{2\sum \nolimits _{i = 1}^n {{p_i}} {y_i}}}{{\sum \nolimits _{i = 1}^n {\left( {{p_i} + {y_i}} \right) } + \lambda }}, \end{aligned}$$

where *n* represents the number of pixels, ${y_i}$ represents the real label of the *i*-th pixel, and ${p_i}$ represents the prediction probability of the *i*-th pixel belonging to the tumor location. To prevent the denominator from being zero, a smoothing factor $\lambda$ is added. Although Dice losses are highly compatible with class-unbalanced data, it is challenging to achieve good segmentation performance using Dice losses alone in network training. Due to the pixel imbalance between tumor and normal tissue in medical images, to segment tumor sites more accurately, we combined the advantages of the two-loss functions to construct a mixed loss function ${L_{\textrm{seg}}}$, which is defined as follows:

13 $$\begin{aligned} {L_{\textrm{seg}}} = \alpha \cdot {L_{\textrm{BCE}}} + \beta \cdot {L_{\textrm{Dice}}}, \end{aligned}$$

where, ${L_{\textrm{BCE}}}$ is the cross-entropy loss, ${L_{\textrm{Dice}}}$ is the Dice loss, $\alpha$ and $\beta$ are the hyperparameters in the mixed loss function, $\alpha ,\beta \in \left[ {0,1} \right]$. In this paper, $\alpha \mathrm{{ = 0}}\mathrm{{.4}}$, $\beta \mathrm{{ = 0}}\mathrm{{.6}}$ are selected.

## Experimental results

In this work, we used three data sets containing different aneurysm diameters to verify the segmentation performance of the WRANet network for three aneurysms of different sizes. To evaluate the proposed model, the segmentation results were compared with U-Net [20], SegNet [13], DeepLabv3+ [14], ResUnet [21], R2U-net [53], Swin-Unet [27], Att-UNet [24], CE-Net [54], and HarDNet-MSEG [55].

### Implementation details and evaluation methods

In this work, we used a combination of Dice and cross-entropy functions as a loss function for the WRANet network, which was implemented based on the Pytorch framework under a GPU server with two Intel(R) Xeon(R) Gold 6226R CPUs. It runs at 2.9 Ghz, has 384 G of RAM, and has two Tesla V100 GPUs with 32GB of RAM. Adam method [56] was used to optimize the parameters of this model, $\beta$1 = 0.9, $\beta$2 = 0.999, eps = 1e−8, weight decay = 5e−4, the initial learning rate was 0.0001, batch size was 4, and iteration was 200 times. We used fivefold cross-validation and a final evaluation of the test set.

To evaluate the segmentation performance of the model, the following indicators are used for evaluation, including Dice coefficient (Dice), intersection over union (IoU), precision (PR), and sensitivity (SE). They are defined as follows:

14 $$\begin{aligned}{} & {} \textrm{Dice }= \frac{{2\mid {{\varvec{R}_{\textrm{gt}}} \bigcap {\varvec{R}_{\textrm{pred}}}\mid }}}{{ \mid {{\varvec{R}_{\textrm{gt}}}}\mid + \mid {{\varvec{R}_{\textrm{pred}}}} \mid }}, \end{aligned}$$

15 $$\begin{aligned}{} & {} {\text {IoU}}=\frac{{\mid {{\varvec{R}_{\textrm{gt}}} \bigcap {\varvec{R}_{\textrm{pred}}}\mid }}}{{ \mid {{\varvec{R}_{\textrm{gt}}}} \bigcup {{\varvec{R}_{\textrm{pred}}}} \mid }}, \end{aligned}$$

16 $$\begin{aligned}{} & {} \textrm{PR} = \frac{\textrm{TP}}{\mathrm{TP + FP}}, \end{aligned}$$

17 $$\begin{aligned}{} & {} \textrm{SE} = \frac{\textrm{TP}}{\mathrm{TP + FN}}, \end{aligned}$$

where, ${\varvec{R}_{\textrm{gt}}}$ represents the tumor region, ${\varvec{R}_{\textrm{pred}}}$ represents the segmentation result predicted by the model, and $\textrm{TP}$, $\textrm{FP}$, $\textrm{FN}$, and $\textrm{TN}$ refer to a true positive, false positive, true negative, and false negative.

### Aneurysm dataset

We follow the principles of the Declaration of Helsinki to conduct our research and have received approval from the Ethics Committee of the Affiliated Hospital of Qingdao University.

We collected 3D-TOF-MRA images of 953 patients with unruptured cystic aneurysm (IAS positive) and 150 regular patients without aneurysm (IAS negative) who underwent physical examination or visited the Affiliated Hospital of Qingdao University from January 2013 to May 2020. After the assessment, exclusion, and screening, 679 patients were identified for the study, including 579 IAS positive (the number of aneurysms was 636) and 100 IAS negative.

Since each patient contained an unbalanced number of sections, we selected five sections from each patient’s image for the experiment. The aneurysm was annotated manually by experienced doctors as the ground truth. The size of the slice was adjusted to $256\times 256$. To verify the segmentation performance of the model for aneurysms of different sizes, we manually selected three different data sets and divided the training set and test set into a 7:3 ratio. Aneurysm images in the training set and test set were not repeated. IAS1 included 89 cases with aneurysms and 20 cases without aneurysms, IAS2 included 290 cases with aneurysms and 50 cases without aneurysms, and IAS3 included 200 cases with aneurysms and 30 cases without aneurysms. The images collected in this paper are from three different equipment manufacturers (Philips, Siemens, and GE), and their details are shown in Table 1.

CVC-ClinicDB [57] is an open polyp dataset consisting of 612 images with a resolution of 384 $\times$ 288, which we cropped to a size of 256 $\times$ 256 and used as input to the model.

**Table 1:** Aneurysm datasets overview

	IAS1	IAS2	IAS3
Size (mm)	Size > 7	3 $\le$ Size $\le$ 7	Size < 3
Number of IAS	93 (14.6%)	329 (51.7%)	214 (33.7%)
Male (age)		245 (36.1%), 62 ± 12	
Female (age)		434 (63.9%), 65 ±11	
Field strength		315 (49.5%), 1.5T	
Field strength		321 (50.5%), 3.0T	
Device (Philips)		190 (29.9%)	
Device (Siemens)		69 (10.8%)	
Device (GE)		377 (59.3%)	

### Results and discussion

In this subsection, first, we compare WRANet and SOTA methods’ segmentation performance. Second, we perform adequate ablation experiments to verify each component’s contribution in WRANet and compare the number of parameters, FLOPs, and inference times. Finally, we validate the generalization performance of the WRANet method with a public dataset.

**Comparison with SOTA models.** In this work, we used three datasets containing different aneurysm diameters and the CVC-ClinicDB dataset to validate the segmentation performance of the WRANet network for three different sizes of aneurysms. To evaluate the performance of the proposed model, all comparison methods use the same training dataset as the proposed method and compare their segmentation results with U-Net [20], SegNet [13], DeepLabv3+ [14], ResUnet [21], R2U-net [53], Swin-Unet [27], Att-UNet [ 24], CE-Net [54], and HarDNet-MSEG [55] for comparison. Our WRANet and most of the above segmentation methods are based on CNN, attention mechanism, and residual structure, while the Swin-Unet model is constructed based on Transformer structure. Table 2 shows the experimental results for different diameter-size aneurysm datasets, and Table 3 shows the results for the CVC-ClinicDB dataset.

**WRANet segmentation performance analysis.** Based on the results in Table 2, our proposed WRANet method outperforms most of the methods on several metrics, indicating our proposed module’s validity. Specifically, by comparing with other Dice, IoU, PR, and SE metrics methods, our method outperformed all comparative methods on the aneurysm dataset. Compared to the baseline method, the U-Net model, Dice, IoU, PR, and SE scores improved by 2.42, 2.42, 5.9, and 4.29%, respectively, when the aneurysm diameter was>7 mm. When the aneurysm diameter was between 3 and 7 mm, Dice, IoU, PR, and SE scores improved by 3.26, 2.48, 8.68, and 5.04%, respectively. When the diameter of the aneurysm was less than 3 mm, Dice, IoU, PR, and SE scores improved by 1.64, 0.39, 1.66, and 1.93%, respectively. This good performance was achieved thanks to the stability of U-Net and the effectiveness of the proposed module, which shows that our method is better segmented and has a lower false alarm rate, revealing that our proposed module is very effective.

**Table 2:** Quantitative comparison of the aneurysm dataset with the SOTA method

Models	IAS1				IAS2				IAS3			
	Dice (%)	IoU (%)	PR (%)	SE (%)	Dice (%)	IoU (%)	PR (%)	SE (%)	Dice (%)	IoU (%)	PR (%)	SE (%)
U-Net (baseline) [20]	76.57	66.54	79.31	76.69	59.38	49.35	64.19	60.13	38.07	30.01	44.71	38.61
SegNet [13]	74.52	63.97	76.87	71.59	48.19	32.90	52.92	37.75	26.78	20.36	25.94	36.28
DeepLabv3+ [14]	68.49	56.93	78.40	68.21	43.15	32.19	41.93	55.86	25.16	17.41	21.38	37.12
ResUnet [21]	76.12	65.32	75.49	78.97	60.14	49.63	62.99	64.38	32.69	24.89	41.49	32.93
R2U-net [53]	72.88	62.58	81.01	75.85	57.54	47.64	72.47	60.04	34.17	28.43	42.76	38.22
Att-UNet [24]	73.64	63.29	73.52	75.13	57.36	46.79	59.01	61.57	35.49	25.97	42.99	38.98
CE-Net [54]	75.45	64.83	77.31	79.15	51.78	40.59	63.07	55.76	36.53	29.38	44.77	35.42
HarDNet-MSEG [55]	68.73	56.39	65.36	75.16	47.82	44.78	52.45	62.45	27.93	20.54	32.20	36.56
Swin-Unet [27]	77.52	66.59	85.16	79.08	61.38	50.59	72.52	62.01	39.03	29.76	45.92	39.58
WRANet	**78**.**99**	**68**.**96**	**85**.**21**	**80**.**98**	**62**.**64**	**51**.**83**	**72**.**87**	**65**.**17**	**39**.**71**	**30**.**40**	**46**.**37**	**40**.**54**

Bold represent the optimal experimental results

**Table 3:** Quantitative comparison of the CVC-ClinicDB dataset with the SOTA method

Models	Dice (%)	IOU (%)	PR (%)	SE (%)
U-Net (baseline) [20]	87.61	80.25	89.46	87.65
SegNet [13]	77.59	66.43	88.44	77.82
DeepLabv3+ [14]	78.26	66.57	84.58	83.33
ResUnet [21]	88.18	80.93	89.04	88.34
R2U-net [53]	87.91	80.41	90.56	88.25
Att-UNet [24]	88.32	81.13	89.23	88.50
CE-Net [54]	88.26	81.36	89.19	88.05
HarDNet-MSEG [55]	87.59	**82**.**08**	89.60	90.38
Swin-Unet [27]	88.35	81.06	89.65	90.72
WRANet	**88**.**89**	81.74	**91**.**32**	**91**.**07**

Bold represent the optimal experimental results

**Table 4:** Based on a comparison between different wavelet variants and RAM modules

Moedls	Dice (%)	IoU (%)	PR (%)	SE (%)
U-Net (baseline)	76.57	66.54	79.31	76.69
+RAM	76.92	67.34	82.06	76.59
+haar	76.29	66.73	83.70	77.01
+db2	77.95	67.94	83.40	77.47
+db3	77.71	68.09	82.95	78.33
+db4	76.97	67.42	83.53	78.08
+db5	77.61	67.91	84.65	77.24
+db6	77.39	67.69	85.03	77.64
+db7	76.94	67.29	84.10	77.47
+sym2	78.23	68.85	84.49	78.57
+sym3	77.18	67.65	84.07	77.57
+sym4	77.68	68.13	84.04	77.62
+sym5	77.63	68.00	82.64	78.70
+sym6	78.72	68.46	84.93	80.31
+sym7	78.65	68.39	84.86	78.52
+bior1.1	78.41	68.66	82.41	79.07
+bior2.2	77.14	67.90	85.18	76.03
+bior3.3	78.48	68.87	83.66	78.85
+bior4.4	78.10	68.67	83.08	78.94
+rbio1.1	77.65	67.97	83.48	78.58
+rbio2.2	77.08	67.25	82.40	78.77
+rbio3.3	75.46	65.83	83.73	75.26
+rbio4.4	78.29	68.73	83.45	79.78
+RAM+sym6+$L_{\textrm{BCE}}$	78.16	68.67	82.97	79.64
+RAM+sym6+$L_{\textrm{Dice}}$	78.63	68.89	84.71	77.97
+RAM+sym6+$L_{\textrm{seg}}$	**78**.**99**	**68**.**96**	**85**.**21**	**80**.**98**

Bold represent the optimal experimental results

To demonstrate the superiority of our method, we have also performed an experimental comparison on the public dataset CVC-ClinicDB. In Table 3, we perform a similar comparison of the CVC-ClinicDB dataset with other methods. Table 3 shows that our method significantly improves the Dice, PR, and SE metrics, with Dice, IoU, PR, and SE scores improving by 1.28, 1.49, 1.86, and 3.42%, respectively. Compared to the baseline method U-Net, the robustness of feature extraction was enhanced by different wavelet basis functions for feature extraction.

To further demonstrate the performance of WRANet, we used nine SOTA models for comparison. Notably, our network still achieves better segmentation performance for aneurysm diameters smaller than 3 mm, demonstrating that the wavelet downsampling module plays a vital role in extracting features and that the RAM module pays more attention to the tumor region and can distinguish to a large extent between the boundaries of tumor and normal tissue. As can be seen in Tables 2 and 3, WRANet achieved the best segmentation performance with Dice, IoU, PR, and SE of 78.99, 68.96, 85.21, and 62.64%, respectively, in the aneurysm dataset IAS2 and 62.64, 51.83, 72.87, and 65.17%, in the aneurysm dataset IAS3 Dice, IoU, PR, and SE were 39.71, 30.40, 46.37, and 40.54% respectively, and in the CVC-ClinicDB dataset, Dice, IoU, PR, and SE were 88.89, 81.74, 91.32, and 91.07%, respectively. However, the DeepLabv3+ network achieved the worst performance on the Dice metric on all datasets.

**Ablation studies.** We conducted ablation studies separately to verify the effectiveness of the loss function and wavelet transform. When performing feature extraction, some feature information is always lost to a greater or lesser extent, and how to reduce the loss of information during feature propagation is the main task considered in this paper. Therefore, we investigate how the wavelet transform affects the network’s performance in feature extraction. As different types of wavelets have different scale functions, which results in different extracted features, which will have a massive impact on the model’s performance. We used wavelet basis functions replacing the maximum pooling layer in the U-Net network to investigate their respective performance, such as haar, db, sym, bior, and rbio. We used U-Net as a baseline model to compare with our proposed WRANet, and the experimental results are shown in Table 4. Since direct observation of the model segmentation result images does not accurately judge the model structure, minor differences are not directly observable using the naked eye. Therefore, we used a series of evaluation metrics to assess the model performance, such as the Dice coefficient, IoU, PR, and SE.

**Table 5:** Complexity comparison of our WRANet against SOTA methods model

Methods	Parameters (M)	FLOPs (B)	Inference time (s)
U-Net [20]	**8**.**6**	0.3	**0**.**2826**
SegNet [13]	29.4	0.6	0.2893
DeepLabv3+ [14]	31.6	0.8	0.3081
ResUnet [21]	13.9	0.5	0.2917
R2U-net [53]	39.1	2.4	0.5863
Att-UNet [24]	34.9	1.1	0.3119
CE-Net [54]	29.0	**0**.**1**	0.3529
HarDNet-MSEG [55]	35.4	0.2	0.4127
Swin-Unet [27]	37.2	2.3	0.5025
WRANet	34.6	1.1	0.3046

Bold represent the optimal experimental results

Table 4 shows different wavelet basis functions as the scores of various indicators of the pooling layer, and wavelet ’sym6’ achieved the highest Dice, IoU, PR, and SE scores. ’Daubechies’ wavelet can improve the performance of the network at low order (’db2’), and the Dice score is 77.95%. However, it will reduce the learning ability of the network with the increase of the order, resulting in a decrease in performance, such as high order ( ’db7’) ’Daubechies’ wavelet has a Dice score of 76.94%. However, the ’Symlets’ wavelet was accompanied by an increase in order (’sym6’), and the performance of the network became better with a Dice score of 78.72%; the lower the order (’sym3 ’, ’sym4’, and ’sym5’), the worse the network performs, with Dice scores of 77.18, 77.68, and 77.63%.The biorthogonal wavelets ’Biorthogonal’ and ’Reversebior’ also improved the segmentation performance. The downsampling process using symmetric wavelets generally performs better than asymmetric wavelets. We choose $L_{\textrm{seg}}$ as the loss function to study wavelet denoising performance. To make the experiment more convincing, we study the influence of $L_{\textrm{BCE}}$ and $L_{\textrm{Dice}}$ on the accuracy of segmentation results, respectively. In addition, we also introduce a RAM module, which can make the feature maps more focused on the target region, making its predictions more closely match the ground truth.

**Fig. 6:**
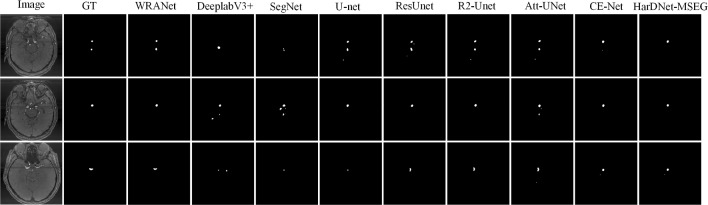
Segmentation visualization results for each SOTA model on dataset IAS1

**Fig. 7:**
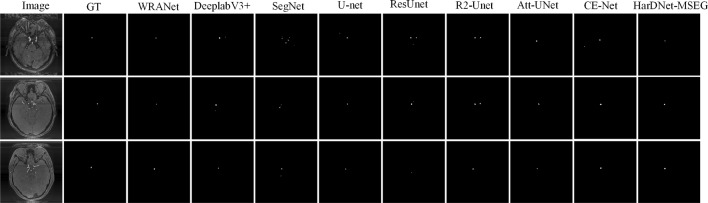
Segmentation visualization results for each SOTA model on dataset IAS2

**Fig. 8:**
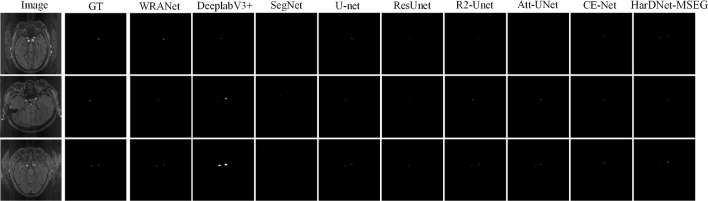
Segmentation visualization results for each SOTA model on dataset IAS3

**Fig. 9:**
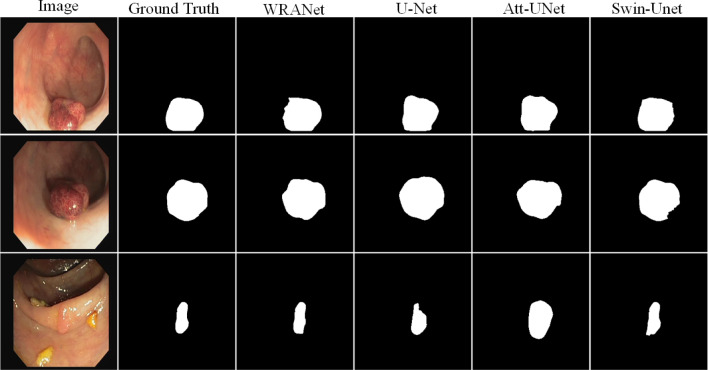
Segmentation visualization results for each SOTA model on dataset CVC-ClinicDB

We validate our proposed method on private and public datasets, respectively. In addition, we found that it is more effective to perform the wavelet downsampling process in the encoder stage because its feature map is more concentrated, which can extract more helpful feature information, remove unnecessary noise, reduce the false positive rate, and improve the network of robustness and segmentation performance.

**Complexity analysis.** We analyzed the number of parameters, FLOPs, and inference time for the WRANet and SOTA models, and the analysis results are shown in Table 5. Although the WRANet method has higher complexity, it has fewer parameters and a shorter inference time than R2U-net, Att-UNet, HarDNet-MSEG, and Swin-Unet, due to its improved performance in aneurysm and polyp segmentation, making it acceptable in our study.

**Visualization of segmentation results.** Figures 6, 7, and 8 show the segmentation visualization results for the IAS1, IAS2, and IAS3 datasets. It is clear that our method yields more accurate results. The first column represents the original image, the second column represents Ground Truth, and the remaining columns are U-Net, SegNet, DeepLabv3+, ResUnet, R2U-net, Att-UNet, CE-Net, HarDNet-MSEG, and WRANet. Visually, our WRANet achieves good segmentation performance while accurately identifying tumor locations. It can be seen in the IAS3 data set that when the aneurysm is small, our method still achieves better performance, while the SegNet, U-Net, ResUnet, and R2-Unet models cannot accurately segment the aneurysm. The proposed model can learn more detailed features from the dataset. Thus, the results show better performance compared to other SOTA models.

Figure 9 represents the visual segmentation results for the CVC-ClinicDB dataset. As shown in Fig. 9, the U-Net, Att-UNet, and Swin-Unet methods have inaccurate boundaries for polyp segmentation, and our WRANet method performs better visually. This indicates that our proposed RAM module and wavelet feature extraction layer play an important role in extracting more complete tumor features and improving tumor segmentation accuracy.

## Conclusion

In this paper, we proposed a wavelet residual attention network WRANet to improve aneurysm segmentation performance. We design a discrete wavelet transform layer (DWT) to replace conventional downsampling operations (max pooling and average pooling) to capture tumor features and remove noise information effectively. We used the RAM module to capture contextual information and fuse multi-scale features, which can recalibrate attention weights to make the network pay more attention to tumor regions, use skip connections to enhance fusion features, and make full use of standard features to improve the generalization performance of the model, which reflects the superiority of attention mechanism and residual connection. In addition, the proposed method overcomes the propagation of high-frequency noise of images in the network and the loss of information to a certain extent. Compared with the standard U-Net and its variants ResUnet, Att-UNet, and R2U-net, the performance of the WRANet network is significantly improved.

The experimental results of image segmentation on the IAS1, IAS2, and IAS3 datasets containing different tumor sizes show that the WRANet network improves the segmentation performance of small tumors and has the potential to help doctors improve the efficiency of diagnosis in clinical practice. It can be seen that the proposed WRANet network is a promising method for medical segmentation. The technique can be easily applied to 3D medical image segmentation in future work.

## Data Availability

The datasets generated during and analyzed during the current study are not publicly available as we have signed a non-disclosure agreement with the hospital to protect patient information, but are available from the corresponding author on reasonable request.
